# High-resolution CT scoring system-based grading scale predicts the clinical outcomes in patients with idiopathic pulmonary fibrosis

**DOI:** 10.1186/1465-9921-15-10

**Published:** 2014-01-30

**Authors:** Keishi Oda, Hiroshi Ishimoto, Kazuhiro Yatera, Keisuke Naito, Takaaki Ogoshi, Kei Yamasaki, Tomotoshi Imanaga, Toru Tsuda, Hiroyuki Nakao, Toshinori Kawanami, Hiroshi Mukae

**Affiliations:** 1 Department of Respiratory Medicine, University of Occupational and Environmental Health, Japan, 1-1, Iseigaoka, Yahatanishiku, Kitakyushu City, Fukuoka 807-8555, Japan; 2 Department of Respiratory Medicine, Steel Memorial Yawata Hospital, 1-1-1, Harunomachi, Yahatahigashiku, Kitakyushu City, Fukuoka 805-8508, Japan; 3 Department of Medicine, Kirigaoka Tsuda Hospital, 3-9-20 Kirigaoka, Kokuraminamiku, Kitakyushu City, Fukuoka 802-0052, Japan; 4 Department of Epidemiology, National Institute of Public Health, Wako, Saitama, Japan, 2-3-6 Minami, Wako-shi, Saitama 351-0197, Japan

**Keywords:** HRCT fibrosis score, UIP pattern, Spirometry, Monitoring methods, Idiopathic pulmonary fibrosis

## Abstract

**Background:**

The 2011 idiopathic pulmonary fibrosis (IPF) guidelines are based on the diagnosis of IPF using only high-resolution computed tomography (HRCT). However, few studies have thus far reviewed the usefulness of the HRCT scoring system based on the grading scale provided in the guidelines. We retrospectively studied 98 patients with respect to assess the prognostic value of changes in HRCT findings using a new HRCT scoring system based on the grading scale published in the guidelines.

**Methods:**

Consecutive patients with IPF who were diagnosed using HRCT alone between January 2008 and January 2012 were evaluated. HRCT examinations and pulmonary function tests were performed at six-month intervals for the first year after diagnosis. The HRCT findings were evaluated using the new HRCT scoring system (HRCT fibrosis score) over time. The findings and survival rates were analyzed using a Kaplan-Meier analysis.

**Results:**

The HRCT fibrosis scores at six and 12 months after diagnosis were significantly increased compared to those observed at the initial diagnosis (p < 0.001). The patients with an elevated HRCT fibrosis score at six months based on a receiver operating characteristic (ROC) curves analysis had a poor prognosis (log-rank, hazard ratio [HR] 2.435, 95% CI 1.196-4.962; p = 0.0142). Furthermore, among the patients without marked changes in %FVC, those with an elevated score above the cut-off value had a poor prognosis (HR 2.192, 95% CI 1.003-4.791; p = 0.0491).

**Conclusions:**

Our data demonstrate that the HRCT scoring system based on the grading scale is useful for predicting the clinical outcomes of IPF and identifying patients with an adverse prognosis when used in combination with spirometry.

## Introduction

Idiopathic pulmonary fibrosis (IPF) is a progressive and generally fatal disease. Several retrospective studies have suggested that the condition is associated with a median survival time of only two to three years after diagnosis [1-5].

The 2011 IPF guidelines provide updated and simplified IPF diagnostic criteria proposed by the ATS/ERS/JRS/ALAT [6]. This may result in HRCT scanning playing a central role in the diagnosis of IPF. According to the guidelines that include major changes in the process of the diagnosis of IPF, the exclusion of other known causes of interstitial lung disease in addition to the detection of the usual interstitial pneumonia (UIP) pattern on high-resolution computed tomography (HRCT) in patients not subjected to surgical lung biopsies (SLBs) is adequate to diagnose the disease.

Furthermore, the 2011 IPF guidelines state that disease progression manifests as worsening respiratory symptoms, worsening pulmonary functions, the presence of progressive fibrosis on HRCT and acute respiratory decline and that monitoring patients with IPF is necessary to detect the development of the disease and proactively identify those with progressive disease. Additionally, pulmonary function tests are considered to be the most standardized approach for objectively monitoring and quantifying disease progression. In clinical practice, it is sometimes difficult to evaluate the progression of the disease based only on a worsening of the pulmonary function due to patient’s non-cooperation.

On the other hand, technological advances in HRCT have brought about decreases in examination times and the ability to obtain clearer images of secondary pulmonary lobules without the need for patient cooperation, unlike spirometry. Therefore, HRCT is an accurate, sensitive and objective technique for evaluating IPF. In addition, physicians often experience patients who exhibit worsening of HRCT findings associated with a poor prognosis in clinical practice. However, the use of regular follow-up with chest HRCT remains controversial in routine clinical practice and the procedure is not currently recommended in clinically stable patients.

The aim of our study was to assess the prognostic value of changes in HRCT findings using a new HRCT scoring system based on the grading scale published in the guidelines.

## Methods

### Study subjects

Our institutional review board approved this retrospective study (approval number H24-174, February 20, 2013), with a waiver of informed consent due to the retrospective study design. All consecutive patients diagnosed with IPF between January 2008 and January 2012 were enrolled in this study. All patients with IPF diagnosed using HRCT alone according to the 2011 IPF guidelines (the presence of an UIP pattern, including all four of the following features: subpleural features, basal predominance, reticular abnormalities, honeycombing with or without traction bronchiectasis) and the absence of features inconsistent with the UIP pattern were included. The patients were also diagnosed based on the exclusion of a possible UIP pattern associated with other known causes of interstitial lung disease, such as chronic hypersensitivity pneumonia, occupational or environmental exposure, connective tissue disease and drug-induced pneumonia. HRCT examinations, pulmonary function tests and serological studies were performed every six months from the initial diagnosis (baseline). Patients with missing or failed inspiratory chest CT scans were excluded from this study. An acute exacerbation was defined as the acute onset of increased dyspnea and hypoxia with progressive infiltrates on HRCT within the preceding 30 days in the absence of infection, pulmonary embolism or cardiac failure [7].

### HRCT assessment and HRCT fibrosis score

HRCT scans were obtained with 1-mm collimation and a 1-mm slice thickness at 10-mm intervals from the lung apices to the bases with the patient in the supine position at full inspiration. Two observers [H.I, K.N.] who were unaware of the clinical data and lung function of the patients (all the HRCT images were assessed in random order) evaluated the data independently. The observers made a subjective assessment of the overall extent of normal attenuation, reticular abnormalities, honeycombing and traction bronchiectasis.

A reticular abnormality was defined as a collection of innumerable areas of small linear opacity [1]. Honeycombing was defined as the presence of a cystic airspace measuring 3–10 mm in diameter, with 1- to 3-mm thick walls [8]. Traction bronchiectasis was defined as irregular bronchial dilatation within the surrounding areas showing parenchymal abnormalities. The morphological criteria on HRCT scans included bronchial dilatation with respect to the accompanying pulmonary artery, a lack of tapering of the bronchi and the identification of bronchi within 10 mm of the pleural surface [8].

The HRCT findings were graded on a scale of 1–4 based on the classification system: 1. normal attenuation; 2. reticular abnormality; 3. traction bronchiectasis; and 4. honeycombing. The assessments of the two observers were averaged. This grading scale and assessed zones were determined based on the previous reports by Ichikado et al. [9,10] with minor changes for this study. The presence of each of the above four HRCT findings was assessed independently in three (upper, middle and lower) zones of each lung. The upper lung zone was defined as the area of the lung above the level of the tracheal carina, the lower lung zone was defined as the area of the lung below the level of the inferior pulmonary vein and the middle lung zone was defined as the area of the lung between the upper and lower zones. The extent of each HRCT finding was determined by visually estimating the percentage (to the nearest 5%) of parenchymal involvement in each zone. The score for each zone was calculated by multiplying the percentage of the area by the grading scale score [1-4]. The six zone scores were averaged to determine the total score for each patient. The highest score was 400 points and the lowest score was 100 points using this calculation method. We named the total score the “HRCT fibrosis score”. The HRCT fibrosis score was recorded at the initial diagnosis and after six and 12 months in a similar manner, and an investigation was conducted regarding the chronological changes in these values.

### Physiological testing

Pulmonary function tests, including spirometry and an assessment of the diffusing capacity of the lungs for carbon monoxide (DL_CO_), were performed using a standardized spirometry procedure [11] on the same day as the HRCT examination. The degree of improvement was defined based on 10% absolute changes in the forced vital capacity (FVC) from the baseline values as “improved (≥ 10% increase),” “stable (< 10% change)” or “worsened (≥ 10% decrease)” using the FVC values measured at six and 12 months after the initial diagnosis. In the present study, disease progression was defined as the presence of acute exacerbation, a ≥10% absolute decrease in the FVC and/or a ≥15% decrease in the %DL_CO_ from the baseline value, as determined according to pulmonary function tests [12].

### Statistical analysis

The mean ± standard deviation (SD) values of the pulmonary function parameters, HRCT fibrosis score and other continuous variables were determined at baseline and at six and 12 months. The paired t-test was performed to evaluate the changes in the variables from the baseline to six and 12 months, respectively. The interobserver variation with respect to the presence/absence of HRCT findings at baseline was evaluated using the kappa statistic based on the diagnosis made prior to the assessment by consensus [13]. The interobserver agreement was categorized as “poor (κ < 0.20),” “fair (0.21 < κ < 0.40),” “moderate (0.41 < κ < 0.60),” “substantial (0.61 < κ < 0.80)” or “almost perfect (0.81 < κ < 1.00).” The interobserver variation regarding the extent of the HRCT findings at baseline and the changes in the extent of honeycombing from baseline to follow-up at six and 12 months were evaluated using Fleiss’s intraclass correlation coefficient (ICC) [14].

Univariate Cox’s proportional hazard models were used to determine the ability of each variable to predict mortality. Additionally, the stepwise multivariate Cox’s proportional hazards model was used for variables found to be significant (p < 0.05) in the univariate model in order to identify more significant variables.

To analyze the changes in the HRCT fibrosis score (ΔHRCT fibrosis score) from baseline to follow-up (after six or 12 months) as a predictor of disease progression (as stated previously) within one year, we used receiver operating characteristic (ROC) curves and the corresponding area under the curve. The cut-off value for the test was selected based on an analysis of the tabular ROC curve data in order to obtain the best possible sensitivity and specificity. Furthermore, the cut-off value was used to investigate whether the presence of an increase, as determined using the cut-off value, after six months or 12 months was related to the overall survival.

The rates of overall survival were estimated using the Kaplan-Meier method and compared using the log-rank test. The patients were divided into groups based on %FVC at six months and the degree of disease progression according to the HRCT fibrosis score, and the overall survival was studied. All statistical analyses were performed using the Statistical Package for Social Sciences (SPSS, version 19). All tests were performed at a significance level of p < 0.05.

## Results

### Patient characteristics

The baseline demographic, clinical and biological characteristics of the participants are summarized in Table 1. All patients were confirmed to be dead/alive as of April 1, 2013. The mean ± SD observation period was 30 ± 18.8 months, and 35 patients (35.7%) died during the observation period, including 17 (17.3%) due to acute exacerbation, 10 (10.2%) due to respiratory failure, four (4.1%) due to cancer and four (4.1%) due to other known causes. The median survival time of all participants was 45.8 months.

**Table 1:** Baseline characteristics and physiology of patients

Patient, No	98
Sex, male/female, No	55/43
Age, year, mean ± SD	70.6 ± 8.0
BMI, kg/m^2^, mean ± SD	23.1 ± 3.4
Smoker (current/former/never)	17/52/29
PaO_2_, Torr, mean ± SD	80.9 ± 15.1
SpO_2_,%, mean ± SD	96.0 ± 2.51
FVC, L, mean ± SD	2.14 ± 0.80
%FVC,%, mean ± SD	71.0 ± 20.8
FEV1.0%,%, mean ± SD	85.1 ± 9.15
DL_CO_, ml/min/mmHg, mean ± SD	9.51 ± 2.83
%DL_CO_,%, mean ± SD	60.0 ± 19.7
KL-6, U/ml, mean ± SD	1319 ± 996
SP-D, ng/ml, mean ± SD	302.2 ± 213.2
LDH, IU/L, mean ± SD	236.2 ± 43.7
MMRC score (0/1/2/3/4)	15/38/24/21/0
HRCT score, mean ± SD	134.2 ± 27.5
Receiving immunosuppressive therapy, No	27

BMI = body mass index, FVC = forced vital capacity, DL_CO_ = diffusing capacity for carbon monoxide, KL-6 = Krebs von den Lungen-6, SP-D = surfactant protein-D, MMRC = modified medical research council.

### Interobserver agreement and correlation

The interobserver agreement with regard to the presence of HRCT findings ranged from substantial to almost perfect (κ value, 0.65-1.00), as did the interobserver agreement regarding the extent of HRCT findings between the two observers ranged [15] (ICC, 0.77-0.91) (Table 2). The interobserver agreement regarding the changes in the extent of honeycombing between the two observers was also almost perfect (ICC, 0.91 and 0.83) (Table 3).

**Table 2:** The distributions of and interobserver agreement between the two observers at baseline

**HRCT findings**	κ value^* ^**(p-value)**	**ICC**^**† **^**(95% CI)**
Normal attenuation	1.00 (< 0.001)	0.91 (0.83-0.95)
Reticular abnormality	0.65 (0.023)	0.83 (0.72-0.91)
Traction bronchiectasis	0.78 (0.031)	0.81 (0.66-0.89)
Honeycombing	0.82 (0.007)	0.77 (0.62-0.87)

^*^The interobserver agreement regarding the presence/absence of HRCT findings between the two observers was assessed by the kappa statistics (κ value).

^†^The interobserver agreement regarding the extent of the HRCT findings between the two observers was analyzed for the square root of the normal attenuation, reticular abnormality, traction bronchiectasis and honeycombing by Fleiss’s ICC.

**Table 3:** Distributions of and interobserver agreement for the changes in the extent of honeycombing from baseline

**From baseline**	**ICC (95% CI)**
at 6 months	0.91 (0.83-0.95)
at 12 months	0.83 (0.68-0.91)

The interobserver agreement regarding the changes in the extent of honeycombing between the two observers were analyzed for the square root of the values at six and 12 months by Fleiss’s ICC.

### Prognostic factors for mortality

The results of the univariate Cox proportional hazard model are shown in Table 4. The %FVC (hazard ratio (HR) 0.968, 95% CI 0.948-0.989; p = 0.002), KL-6 (HR 1.000, 95% CI 1.000-1.001; p = 0.028) and modified Medical Research Council (MMRC) score (HR 1.659, 95% CI 1.111-2.478; p = 0.013) were identified to be significantly predictive of survival. In this analysis, the HRCT fibrosis score at baseline was not found to be significantly related to the prognosis. Meanwhile, the multivariate Cox proportional analysis demonstrated the %FVC (HR 0.972, 95% CI 0.958-0.986; p = 0.037) and MMRC score (HR 1.240, 95% CI 1.005-2.475; p = 0.043) to be significant factors predicting the prognosis of patients with IPF.

**Table 4:** Results the univariate and multivariate Cox proportional hazard models of independent predictors of mortality

**Univariate Cox proportional hazard model**		
**Variable**	**p-value**	**HR (95% Cl)**
Age	0.219	1.023 (0.985 to 1.062)
BMI	0.292	0.937 (0.829 to 1.058)
PaO_2_	0.193	0.982 (0.955 to 1.009)
%FVC	0.002	0.968 (0.948 to 0.989)
KL-6	0.028	1.000 (1.000 to 1.001)
MMRC score	0.013	1.659 (1.111 to 2.478)
HRCT score	0.268	1.007 (0.995 to 1.019)
**Multivariate Cox proportional hazard model**		
%FVC	0.037	0.972 (0.958 to 0.986)
KL-6	0.100	1.000 (1.000 to 1.001)
MMRC score	0.043	1.240 (1.005 to 2.475)

BMI = body mass index, FVC = forced vital capacity, KL-6 = Krebs von den Lungen-6, MMRC = modified medical research council.

### Time-dependent changes in %FVC and the stratified survival analysis

**Figure 1:**
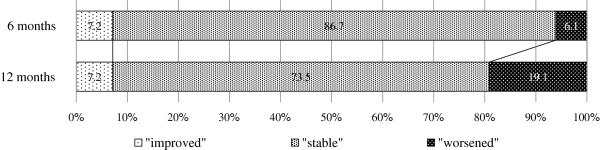
**The results of the categorical analysis based on the changes in the FVC from baseline.** The %FVC declined with time. Improvement ratings were defined based on a 10% absolute change as “improved” for a ≥ 10% increase, “stable” for a < 10% change and “worsened” for a ≥ 10% decrease.

The %FVC values “improved” at six and 12 months after the initial diagnosis in seven and seven patients (7.2% and 7.2%), remained “stable” in 85 and 73 patients (86.7% and 73.5%) and “worsened” in six and 18 patients (6.1% and 19.1%), respectively, thus indicating worsening of %FVC over time (Figure 1).

**Figure 2:**
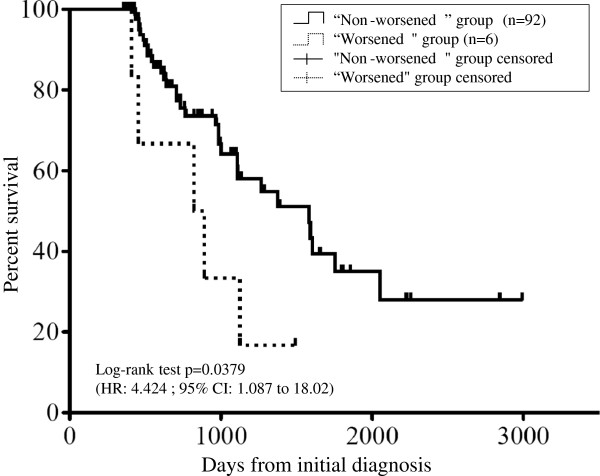
**The results of the Kaplan-Meier analysis of the overall survival.** The overall survival of the “worsened” status group at six months was significantly worse than that of the “non-worsened” status group at six months (p = 0.0379).

The positive and negative predictive values with the ratings at six months for the prediction of those at 12 months are shown in Table 5. In this analysis, the “worsened” at six months was still present at 12 months at a relatively high rate (positive predictive value: 66.7% (4/6)), and the “non-worsened” status group at six months remained relatively stable at 12 months with a high rate (negative predictive value: 83.7% (77/92)). Additionally, the overall survival analysis of the patients divided into two groups according to the presence or absence of a “worsened” status at six months showed that the “worsened” status group had a poorer prognosis (log-rank test, HR 4.424, 95% CI 1.087-18.02; p = 0.0397) (Figure 2). The median survival rates of the “non-worsened” and “worsened” status groups were 52.7 and 28.6 months, respectively.

**Table 5:** Positive and negative predictive values at six months for predicting the ratings at 12 months

		**at 12 months**		
		worsened^*^	**Non-worsened**^**†**^	
at 6 months	worsened	4 (66.7%)	2 (33.3%)	6
	Non-worsened	15 (16.3%)	77 (83.7%)	92
		19	79	98

^*^Worsened was defined based on 10% absolute decreasing changes in the FVC from baseline.

^†^Non-worsened indicated improved (≥ 10% increase) and stable (< 10% change) status.

### Time-dependent changes in the HRCT fibrosis score and the stratified survival analysis

**Figure 3:**
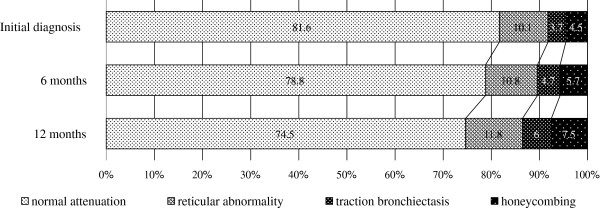
**The changes in the extent of each HRCT finding after the initial diagnosis.** The ratios of the radiological findings of reticular abnormalities, traction bronchiectasis and honeycombing increased over time, while the ratio of normal attenuations decreased.

**Figure 4:**
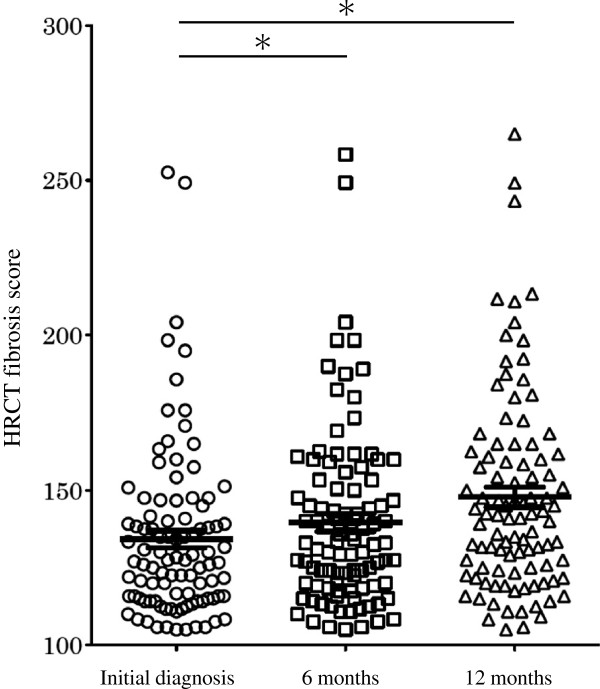
**The changes in the total HRCT fibrosis score after the initial diagnosis.** The HRCT fibrosis score significantly increased at six and 12 months compared to the initial diagnosis (*p < 0.001).

**Figure 5:**
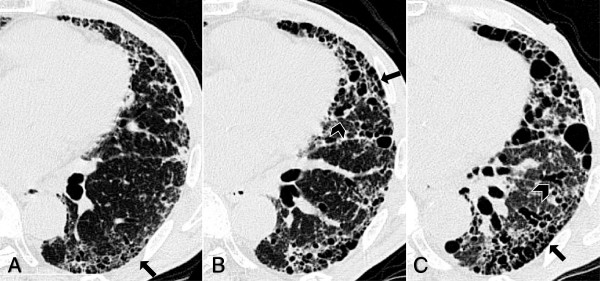
**High-resolution CT findings of the patient with an adverse prognosis.** The patient was a 78-year-old man with IPF. HRCT scan at the level of lower left lobe shows fibrosing changes from the baseline **(A)** at six **(B)** and 12 months **(C)**. HRCT images at diagnosis of IPF show subpleural predominant interstitial fibrosis, traction bronchiectasis (arrowheads) and honeycombing (arrows). The overall HRCT fibrosis score at the baseline, six and 12 months were 151.3, 162.5 and 185.8, respectively. This patient died 20 months after initial diagnosis.

The overall extent of pulmonary parenchymal abnormalities on the initial diagnosis and at six and 12 months is summarized in Figure 3. In line with the changes observed at six and 12 months after the initial diagnosis, the extent of normal attenuation was reduced, while that of reticular abnormalities, honeycombing and traction bronchiectasis was increased. Furthermore, the average HRCT fibrosis score increased over time, with scores of 134.2, 139.6 and 147.8 at baseline and six and 12 months, respectively (p < 0.001) (Figures 4 and 5).

The area under the ROC curve for the ΔHRCT fibrosis score was used to significantly classify patients with regard to disease progression at six and 12 months (0.691; 95% CI 0.518-0.871; p = 0.033, 0.712; 95% CI 0.542-0.881; p = 0.024, respectively). The optimal cut-off value for the ΔHRCT fibrosis score was 6.7 and 13.5 at six and 12 months, respectively, based on the Youden index, with a sensitivity and specificity of the respective diagnostic performance for predicting disease progression of 0.64 (95% CI 0.31-0.89) and 0.72 (95% CI 0.60-0.81) at six months and 0.82 (95% CI 0.48-0.98) and 0.64 (95% CI 0.52-0.75) at 12 months, respectively.

**Figure 6:**
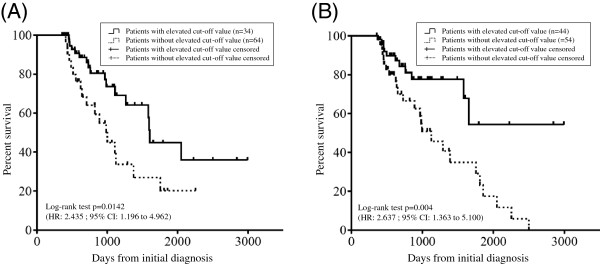
**The difference in the overall survival whether the cut-off value elevated or not.** The overall survival of patients with an elevated value was significantly worse than that of patients with value below the cut-off. The cut-off values of the ΔHRCT fibrosis score at six **(A)** and 12 **(B)** months were 6.7 and 13.5, respectively.

Patients with an elevated ΔHRCT fibrosis score above the cut-off value at six (HR 2.435, 95% CI 1.196-4.962; p = 0.0142) (Figure 6A) and 12 months (HR 2.637, 95% CI 1.363-5.100; p = 0.004) (Figure 6B) compared to that observed at baseline had a significantly worse prognosis than those with lower scores.

### Survival analysis using a combination of the HRCT fibrosis score and %FVC

**Figure 7:**
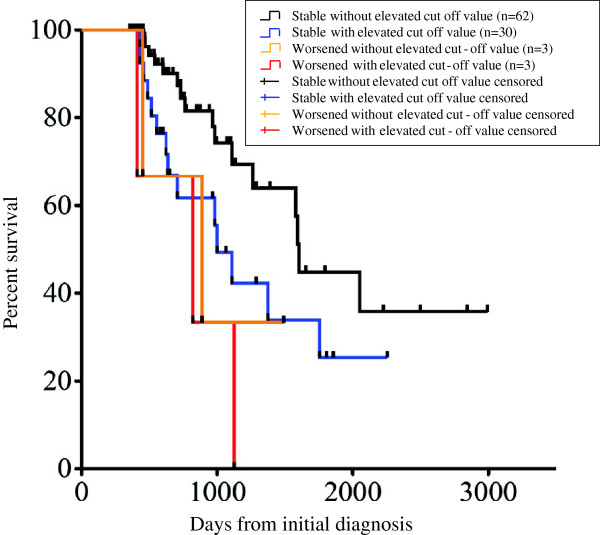
**The results of a comparison of the overall survival between the IPF patient groups.** Among the patients in whom the %FVC was of “non-worsened” status at six months, the patients with an increased HRCT fibrosis score above the cut-off value demonstrated a significantly poorer prognosis, with a decreased overall survival, than those without such an increase (HR 2.192, 95% CI 1.003-4.791; p = 0.0491).

At six months, the patients with increased HRCT fibrosis scores above the cut-off value in addition to a “worsened” status regarding the %FVC had a clearly poorer prognosis (HR 28.47, 95% CI 2.649-306.5; p = 0.0057). Furthermore, in the comparison of the overall survival based on whether the HRCT fibrosis score was increased above the cut-off value among the patients with a “non-worsened” status regarding the %FVC at six months, those with an increased HRCT fibrosis score above the cut-off value demonstrated a significantly poorer prognosis (HR 2.192, 95% CI 1.003-4.791; p = 0.0491) (Figure 7).

## Discussion

In the present study, we first clarified that changes over time in HRCT findings in combination with the %FVC predict the prognosis of patients with IPF. In addition, our data demonstrated that the new HRCT scoring system is helpful for identifying patients with an adverse prognosis when used in combination with pulmonary function examinations. These results indicate that the new HRCT scoring system is an appropriate and subjective method for monitoring IPF patients.

In the present study, the %FVC was found to be a baseline factor predicting the prognosis of patients with IPF. In addition, the patients with a decline of ≥ 10% in the absolute value of FVC six months after the initial diagnosis (the “worsened” status group) had a poor prognosis in this study. Furthermore, these patients demonstrated a tendency toward further declines in the %FVC over the subsequent six months and to have a worse prognosis. On the other hand, the vast majority of the patients in the present study (93.9%) exhibited an “improved” or “stable” status six months after the initial diagnosis and tended to not show any changes in the %FVC over the following six months. Changes in the %FVC are an indicator of disease progression as a surrogate endpoint for overall survival [15-17]; therefore, pulmonary function examinations are commonly used to monitor patients with IPF. However, the clinical course of IPF varies widely [18,19], and some patients may exhibit a sudden decrease in their pulmonary function [20]. The variety in the clinical course of patients with IPF makes it difficult to identify those with a poor prognosis based on the results of pulmonary function tests alone, and it is therefore necessary to establish a proper monitoring method enabling clinicians to identify the patients most likely to have a poor prognosis from a different perspective.

Previous reports regarding the relationship between HRCT findings and survival rates in IPF patients have been published [21-23]. The CT visual score [24] and fibrotic score [2,25] determined using various software tools [26] to evaluate findings of reticular abnormalities and honeycombing are useful for assessing the prognosis of IPF. Furthermore, some reports [27,28] have evaluated changes in HRCT findings in patients with IPF over time using a scoring system. In the present study, we used the new HRCT scoring system-based grading scale. The new HRCT scoring system was designed to reflect all findings of the UIP pattern described in the 2011 IPF guidelines. The grading scale reflects the progression of pulmonary fibrosis. Additionally, we graded the HRCT findings in order of priority from honeycombing to traction bronchiectasis, reticular abnormalities and normal attenuation. Furthermore, we set the monitoring period at six-month intervals in order to accurately investigate the appropriate duration for monitoring disease progression in patients with IPF.

The HRCT fibrosis score on the initial diagnosis was not found to be a factor predicting the prognosis in this study, although the extent of changes in the HRCT fibrosis score within at least the first six months after the initial diagnosis did reflect the prognosis, and acceleration in the findings of pulmonary fibrosis on radiology had an impact on the final outcome. In terms of the extent of acceleration of fibrosis, the number of fibroblastic foci has previously been shown to have an impact on the prognosis in pathological studies [5,29,30] and is an important factor associated with the clinical state of IPF. Additionally, the combined evaluation of radiological assessment within the first six months after the initial diagnosis with a change in %FVC facilitated the extraction of patients with a poor prognosis, who were otherwise hidden within the group with stable %FVC values. In other words, among the patients with IPF diagnosed using only HRCT, the %FVC was found to be a baseline factor indicating the prognosis, and pulmonary function examinations (as a monitoring method) in combination with the assessment of HRCT findings were useful for inferring the detailed prognosis.

From the standpoint of early detection of concomitant lung cancer [31,32], it is important to avoid overlooking small lung cancer lesions, even on routine HRCT (instead of incremental CT). Furthermore, in line with the 2011 revisions to the guidelines, it is anticipated that diagnosing IPF using only HRCT will become more common in the future, as this modality also facilitates the determination of responsiveness to treatment [33], and it is believed that HRCT examinations will continue to play a pivotal role in the diagnosis and management of IPF.

There are several limitations associated with this study. First, the design study was retrospective, and the treatments given to the patients were not identical, which may have influenced the assessments of the prognosis. Second, only 19.1% of the patients were allocated to the group in which a decrease of ≥ 10% in the absolute value of FVC was observed 12 months after the initial diagnosis (the “worsened” status group). This group is considerably smaller than that observed in previous studies, including a proportion of 36.9% in a Japanese study of pirfenidone [34] and 41.9% in a study of etanercept [35]. The fact that all patients who died within one year of the initial diagnosis were eliminated in the present study may be a further limitation. Third, the diagnosis of IPF was restricted to patients in whom the UIP pattern was diagnosed using HRCT and did not include those with a possible UIP pattern. In other words, IPF patients requiring pathological consideration were not included, and the study therefore does not reflect the entity of IPF as a whole. Fourth, this work was carried out jointly across multiple facilities, restricted to Japanese IPF patients. For this reason, when interpreting the results, it is necessary to consider potential racial selection bias, etc. Finally, this study was primarily based on HRCT findings obtained using a visual score, with good interobserver variation for the assessment of the presence/absence of each HRCT finding. However, among the various radiological factors, honeycombing has previously been reported [36] to have an insufficient rate of concordance, and radiological changes in this feature over a one-year period are small; thus, it is necessary to pay attention to inaccuracies in the manual scoring system used for the radiological CT examinations.

## Conclusions

The HRCT scoring system-based grading scale is useful for inferring the prognosis in patients with IPF and, in particular, facilitates the extraction of patients with a poor prognosis who cannot be identified using only pulmonary function examinations. These findings have the potential to improve the day-to-day treatment of IPF patients, including the ability to determine the correct time at which to initiate treatment, including the administration of new agents such as pirfenidone [37] and BIBF1120 [38] as well as lung transplantation.

## Abbreviations

IPF: Idiopathic pulmonary fibrosis; UIP: Usual interstitial pneumonia; HRCT: High-resolution computed tomography; SLBs: Surgical lung biopsies; DLCO: Diffusing capacity for carbon monoxide; FVC: Forced vital capacity; SD: Standard deviations; ROC: Receiver operating characteristic; HR: Hazard ratio; MMRC: Modified medical research council.

## Competing interests

The authors declare that they have no competing interests.

## Authors’ contributions

KO, HI and KYat made substantial contributions to the conception and design of the study. KO, TO, TI and TT acquired the data. TO, KN and HN analyzed and interpreted the data. KO, HI, KYat, KYam, TK and HM participated in drafting the article and critically revising it for important intellectual content. All authors have read and approved the final manuscript.
